# Joining COPE: Opportunities and benefits for the Iranian Journal of Basic Medical Sciences

**DOI:** 10.22038/ijbms.2023.76381.16530

**Published:** 2024

**Authors:** Leila Arabi, Ali Roohbakhsh, Bizhan Malaekeh-Nikouei, Bibi Sedigheh Fazly Bazzaz

**Affiliations:** 1 Nanotechnology Research Center, Pharmaceutical Technology Institute, Mashhad University of Medical Sciences, Mashhad, Iran (Assistant Editor); 2 Pharmaceutical Research Center, Pharmaceutical Technology Institute, Mashhad University of Medical Sciences, Mashhad, Iran (Assistant Editor); 3 Biotechnology Research Center, Pharmaceutical Technology Institute, Mashhad University of Medical Sciences, Mashhad, Iran (Editor–in–Chief)

## Introduction

In our 2023 editorial, we mentioned that we are in the process of becoming a member of the Committee on Publication Ethics (COPE). COPE has been supporting members from all academic fields globally for over 25 years. According to Elsevier, COPE is “a forum for editors of peer-reviewed journals to discuss issues related to the integrity of the scientific record” (1). As a general rule, COPE helps journal editors handle ethical issues in publication. 

In May 2021, the Iranian Journal of Basic Medical Sciences (IJBMS) applied for COPE membership. Here, we are pleased to announce that on August 24^th^, 2023, our membership application was approved.

Consequently, IJBMS details have been added to the members list on the COPE website. We recently added the COPE logo to the homepage of our website. The COPE logo in our journal indicates that IJBMS upholds the highest ethical standards based on the COPE guidelines. Moreover, being a member of COPE confirms and ensures that IJBMS intends to act according to COPE’s core practices. In this regard, IJBMS provided more detail on policies relating to misconduct issues. IJBMS adheres to ICMJE and COPE guidelines and outlines the journal’s policies. 

By joining COPE, all IJBMS editors, editorial board members, and journal or publishing colleagues would benefit from accessing all the free and member-only resources on the COPE website. Table 1 demonstrates some of the provided resources.

In addition to the mentioned resources on the COPE website, the “COPE Membership Handbook” provides guidance on the range of benefits available to COPE members in order to maintain the highest ethical standards in research publishing. All of our colleagues and audience are welcome to read this handbook:

https://publicationethics.org/cope-member-handbook

It is worth mentioning that, following COPE membership, our journal has announced its position on the use of artificial intelligence. IJBMS guideline is in agreement with the COPE position statement on the use of Artificial Intelligence (AI) in all steps of manuscript preparation: “Authors are fully responsible for the content of their manuscript, even those parts produced by an AI tool, and are thus liable for any breach of publication ethics.” We encourage our readers to see the following link for more information regarding the COPE position statement on Authorship and AI tools:

https://publicationethics.org/cope-position-statements/ai-author

Lastly, we would like to take this opportunity to express our gratitude to our colleagues for their continued hard work and dedication to IJBMS, as well as to our reviewers for their invaluable assistance. Their contribution is essential for improving our journal’s quality and credibility. In addition, we would like to thank all authors for choosing to publish with IJBMS. Their contribution to the advancement of basic medical research is highly appreciated.

During these difficult times, we wish our readers and colleagues across the globe a healthy, safe, and peaceful NEW YEAR. We will strive to continuously enhance the quality of our services for our audience community in 2024.

**Table 1:** Some of the free and member-only resources on the COPE website

Take part in webinars on hot topics in publication ethics
Online forums where members submit a case they’re currently dealing with for discussion
Access over 500 previous cases our forums have considered and the advice given
Free attendance at COPE’s annual seminars and workshops worldwide
10 eLearning modules covering various publication ethics issues
Review the journal publication ethics standards using our Journal Audit tool
Get advice on ethical issues between Forum meetings from the COPE Council
Benefit from the sample letters to help reviewers and authors about potential misconduct
Access and download the flowcharts and Core Practice guidelines
